# Characterizing Moral Injury and Distress in US Military Surgeons Deployed to Far-Forward Combat Environments in Afghanistan and Iraq

**DOI:** 10.1001/jamanetworkopen.2023.0484

**Published:** 2023-02-23

**Authors:** Madeline Y. Ryu, Matthew J. Martin, Alexander H. Jin, Holly K. Tabor, Sherry M. Wren

**Affiliations:** 1 Stanford University School of Medicine, Stanford, California; 2 Division of Trauma and Acute Care Surgery, Department of Surgery, Los Angeles County and USC Medical Center, Los Angeles, California; 3 Stanford Center for Biomedical Ethics, Department of Medicine, Stanford University, Stanford, California; 4 Surgical Service, Palo Alto Veterans Health Care System, Palo Alto, California; 5 Department of Surgery, Stanford University School of Medicine, Stanford, California

## Abstract

**Importance:**

Moral injury and distress (MID), which occurs when individuals have significant dissonance with their belief system and overwhelming feelings of being powerless to do what is believed to be right, has not been explored in the unique population of military surgeons deployed far forward in active combat settings. Deployed military surgeons provide care to both injured soldiers and civilians under command-driven medical rules of engagement (MROE) in variably resourced settings. This practice setting has no civilian corollary for comparison or current specific tool for measurement.

**Objective:**

To characterize MID among military surgeons deployed during periods of high casualty volumes through a mixed-methods approach.

**Design, Setting, and Participants:**

This qualitative study using convergent mixed methods was performed from May 2020 to October 2020. Participants included US military surgeons who had combat deployments to a far-forward role 2 treatment facility during predefined peak casualty periods in Iraq (2003-2008) and Afghanistan (2009-2012), as identified by purposeful snowball sampling. Data analysis was performed from October 2020 to May 2021.

**Main Outcomes and Measures:**

Measure of Moral Distress for Healthcare Professionals (MMD-HP) survey and individual, semistructured interviews were conducted to thematic saturation.

**Results:**

The total cohort included 20 surgeons (mean [SD] age, 38.1 [5.2] years); 16 (80%) were male, and 16 (80%) had 0 or 1 prior deployment. Deployment locations were Afghanistan (11 surgeons [55%]), Iraq (9 surgeons [45%]), or both locations (3 surgeons [15%]). The mean (SD) MMD-HP score for the surgeons was 104.1 (39.3). The primary thematic domains for MID were distressing outcomes (DO) and MROE. The major subdomains of DO were guilt related to witnessing horrific injuries; treating pregnant women, children, and US soldiers; and second-guessing decisions. The major subdomains for MROE were forced transfer of civilian patients, limited capabilities and resources, inexperience in specialty surgical procedures, and communication with command. Postdeployment manifestations of MID were common and affected sleep, medical practice, and interpersonal relationships.

**Conclusions and Relevance:**

In this qualitative study, MID was ubiquitous in deployed military surgeons. Thematic observations about MID, specifically concerning the domains of DO and MROE, may represent targets for further study to develop an evaluation tool of MID in this population and inform possible programs for identification and mitigation of MID.

## Key Points

**Question:**

What are the factors associated with moral injury and distress (MID) in deployed military surgeons?

**Findings:**

In this pilot qualitative study of 20 deployed military surgeons, primary domains for MID were distressing outcomes (eg, witnessing horrific injuries; treating pregnant women, children, and US soldiers; and second-guessing decisions) and medical rules of engagement (eg, forced transfer of civilian patients, limited capabilities and resources, inexperience in specialty surgical procedures, and communication with command).

**Meaning:**

These findings suggest that there is a need to develop evaluation tools and mechanisms to mitigate MID unique to the military health care population.

## Teaser

This qualitative study examines moral injury and distress among US military surgeons deployed to Iraq and Afghanistan during periods of high casualty volumes through a mixed-methods approach.

## Introduction

Moral injury and moral distress are topics of increasing interest in the medical realms of both civilian and military settings.^1,2,3,4,5^ Moral distress is characterized as “overwhelming feelings of being powerless to do what is believed to be right,”^4^ and moral injury is an event that “generates significant dissonance with the individual’s belief system and worldview.”^6^ Current research on moral injury and distress (MID) has been focused on combat soldiers and civilian health care workers. Most recently, MID has been examined in the context of health care workers during the COVID-19 pandemic.^1,2,3,4,5,7,8^

Health care workers at greatest risk for MID include those who work with patients who are either likely to die (eg, those with cancer or burns) or are from a vulnerable population (eg, children and individuals with disabilities). These can include perceived unsupportive leadership, emotional or psychological consequences of decisions, or inability to meet patient or family needs that can have a profound impact on health care worker well-being.^2,7,9,10,11,12,13^ There is evidence that MID is substantially higher in health care professionals who have considered leaving or have actually left a position compared with those who have not considered resignation.^5^ The concept of moral repair via psychological support, acknowledgment, and support from leadership has been advanced during the recent COVID-19 crisis as a means by which to mitigate the effects of moral injury on hospital staff.^14^ Continued investigations of MID in physicians is vital to ensure physicians’ well-being.

Posttraumatic stress disorder (PTSD) is prevalent among veterans and active duty military and is associated with increased risk of suicide and other negative health behaviors.^15^ MID may also contribute substantially to the insidious and long-lasting mental trauma of combat soldiers.^1,16,17,18^ Physicians often misdiagnose MID as PTSD in soldiers, even though the causes and symptoms of MID are different.^1,19,20^ In PTSD, fear and anxiety are primary features of psychological distress, whereas patients with MID report guilt, shame, and difficulties with forgiveness as main sources of psychological impairment.^2^ Investigations have focused on combat experiences and not military health care service during combat operations.

Despite the growing interest in MID in civilian health care professionals and combat soldiers, there has been little to no research at the intersection of these populations—that is, military health care professionals, specifically surgeons working in active combat or other forward operational settings. MID may be different in military surgeons as a result of unique stressors, obligations, and inability to leave their position. Therefore, our pilot study aims to assess MID in military surgeons who had deployed to far-forward environments in Iraq and Afghanistan during peak casualty times.

## Methods

We performed a convergent, mixed-methods, qualitative study using responses to preinterview survey questions, followed by an interview process via Zoom.^21^ The study was approved by the Stanford University institutional review board, and all participants provided oral informed consent. This study followed the Consolidated Criteria for Reporting Qualitative Research (COREQ) reporting guideline.

### Recruitment

Participants were recruited through purposeful snowball sampling through authors’ personal contacts (S.M.W. and M.J.M.). Following initial interviews, each surgeon was asked to recommend others. We recruited until thematic saturation was reached. All participants were military surgeons who had deployed to a role 2 military treatment facility, defined as an environment close to combat action with basic resuscitative and surgical capabilities but little to no diagnostic tools or imaging technology,^22^ in Operation Iraqi Freedom (OIF) and Operation Enduring Freedom (OEF) during peak casualty periods (2003-2008 for OIF^23^ and 2009-2012 for OEF^24^) (eTable 1 and eTable 2 in Supplement 1).

### Data Collection

#### Quantitative Data

The preinterview survey included demographic questions and the Measure of Moral Distress for Healthcare Professionals (MMD-HP) tool of 27 statements. The MMD-HP uses a scale of 0 to 4 for both frequency and degree of distress summed for a composite score, which can range from a minimum of 0 to a maximum of 432, with higher scores indicating greater distress.^25^

#### Qualitative Data

Participants were invited to participate in individual, semistructured interviews, conducted from May to October 2020. A single investigator (M.Y.R.) interviewed all participants. Interview guide questions were developed from Koenig’s theoretical framework, which explores the continuum from an event that transgress a moral code with the interplay of psychological and religious or spiritual influences on the internal ethical conflict and potential negative clinical outcomes.^20^ Interviews were audio-recorded and transcribed verbatim by the interviewer. All audio recordings were deidentified before analysis, coded as a participant identification number, and anonymously stored in Health Information Portability and Accountability Act–compliant cloud storage.

### Statistical Analysis

Data analysis was performed from October 2020 to May 2021. Interviews were analyzed deductively, with new codes added in an inductive fashion. An initial codebook was developed from a combination of preexisting codes adapted from Koenig’s framework and additional codes while analyzing the interviews.^20^ Dedoose analytic software version 8.0.35 (SocioCultural Research Consultants, LLC) was used for qualitative analysis.^26^ Each transcript was coded independently by 2 coders (M.Y.R. and A.H.J.), who then finalized the codebook via a comparative approach throughout the process.^27^ Interrater reliability tests were conducted to check for consistency of code application process (κ = 0.73).

## Results

The total study cohort included 20 military surgeons (mean [SD] age, 38.1 [5.2] years); 16 (80%) were male, and 16 (80%) had 0 or 1 prior deployment. Ten of 20 (50%) had never deployed before the deployment in question, and only 5 (25%) were above officer rank O-4 (Major or Lieutnant Commander, depending on service branch; physicians’ initial rank at completion of residency) during the deployment. The majority (16 of 20 surgeons [80%]) were early career surgeons with less than 8 years postresidency experience. Deployment locations were Afghanistan (11 surgeons [55%]), Iraq (9 surgeons [45%]), or both locations (3 surgeons [15%]). Specific demographic information collected is listed in Table 1. The mean (SD) interview length was 51.3 (16.3) minutes (range, 35-98 minutes).

**Table 1.** Surgeon Characteristics and Demographics

Characteristic	Surgeons, No. (%) (N = 20)
Officer rank^a^	
O-4 (Major or Lieutenant Commander)	15 (75)
O-5 (Lieutenant Colonel or Commander)	2 (10)
O-6 (Colonel or Captain)	3 (15)
Age, y	
≤35	5 (25)
36-40	11 (55)
41-52	4 (20)
Time after residency that deployment took place, y	
<1	6 (30)
1-8	10 (50)
>8	4 (20)
Deployment length, mo	
≤5	9 (45)
6-8	6 (30)
>8	5 (25)
Deployments before this specific deployment (OEF or OIF), No.	
0	10 (50)
1	6 (30)
2	2 (10)
3	2 (10)
Other surgeons at role 2, No.	
0-1	9 (45)
2-3	9 (45)
>3	2 (10)
Sex	
Male	16 (80)
Female	4 (20)
Combat operation	
OEF	11 (55)
OIF	9 (45)
Both OEF and OIF	3 (15)

Abbreviations: OEF, Operation Enduring Freedom; OIF, Operation Iraqi Freedom.

^a^ Titles vary depending on branch of service.

### Quantitative Findings

Individual MMD-HP scores are reported in the Figure. The mean (SD) MMD-HP score was 104.1 (39.3). The highest score reported was 179, and the lowest was 37. The most highly scored statements in the MMD-HP survey questionnaire concerned patient suffering or receiving nonoptimal care because of a lack of resources, administrative barriers, practitioner continuity, and team communication. The lowest scored statements were items that may not apply to the deployed environment, such as litigation or difficulty with patient family members (Table 2). These statements were explored in further detail during the interviews.

**Figure.**
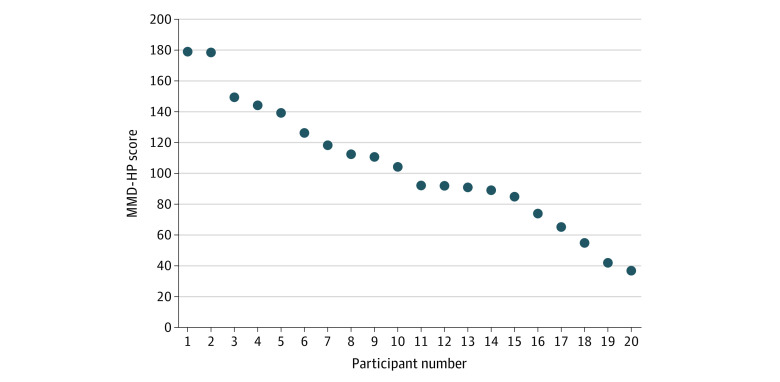
Measure of Moral Distress for Healthcare Professionals (MMD-HP) Survey Tool Graph shows overall composite MMD-HP score for each participant.

**Table 2.** Measure of Moral Distress for Healthcare Professionals Score Given for Each Statement

Measure of Moral Distress for Healthcare Professionals statements	Score, median (IQR)
Watched patient suffer because of a lack of practitioner continuity	8.5 (3.3-16.0)
Was unable to provide optimal care due to pressures from administrators or environment to reduce costs and save resources	7.5 (4.0-11.3)
Experienced compromised patient care due to lack of resources, equipment, or bed capacity	6.0 (4.0-11.3)
Was required to work with other health care team members who are not as competent as patient care requires	5.0 (3.0-11.3)
Witnessed low quality of patient care due to poor team communication or team training	4.0 (1.3-6.0)
Experienced lack of administrative action or support for a problem that is compromising patient care	4.0 (4.0-9.0)
Had excessive documentation requirements that compromise patient care	4.0 (4.0-9.0)
Continued to provide aggressive treatment for someone who will die regardless of treatment	4.0 (1.0-5.5)
Was required to care for patients whom I feel is not qualified for care	4.0 (1.0-8.3)
Gave “false hope” to a patient	4.0 (1.3-11.3)
Was pressured to avoid taking action when I learn that a physician, nurse, or other team colleague has made a error and does not report it	3.5 (1.0-6.0)
Followed the insistence to continue aggressive treatment even though I believe it is futile	3.0 (1.0-5.5)
Felt pressured to order or carry out orders for what I consider to be unnecessary or inappropriate tests and treatments	3.0 (1.0-4.0)
Participate in a team that gives inconsistent messages to patient	3.0 (1.0-4.0)
Felt unsafe or bullied among my own colleagues	3.0 (0.0-4.0)
Worked within power hierarchies, in teams or units that compromise patient care	2.0 (0.3-4.0)
Participate in care that causes unnecessary suffering or does not relieve pain	2.0 (1.0-4.0)
Witnessed a violation of a standard of practice or a code of ethics and not feel sufficiently supported to report the violation	1.5 (0.0-4.0)
Felt required to overemphasize tasks and productivity or quality measures at the expense of patient care	1.0 (0.0-4.0)
Was required to care for more patients than I can safely care for	1.0 (0.0-2.3)
Feared retribution if I speak up	1.0 (1.0-4.0)
Ignore situations where patients not given adequate info for informed consent	1.0 (0.0-3.0)
Be required to care for patients who have unclear treatment plans or lack goals of care	1.0 (0.0-3.8)
Participate in care that I do not agree with but do so because of fear of litigation	1.0 (0.0-3.8)
Worked with team members who do not treat vulnerable or stigmatized patients with dignity and respect	1.0 (0.0-4.0)
Follow family request to not discuss patient’s prognosis	0.5 (0.0-3.0)
Was required to work with abusive patients or family members who are compromising quality of care	0.0 (0.0-2.0)

### Qualitative Findings

Interview analysis found that many surgeons felt that they were required to work with team members who did not have the requisite skills or experience and that there was poor team cohesion and communication. Analysis of the semistructured interviews revealed 2 main domains: distressing outcomes and medical rules of engagement (MROE). Within each domain, we identified 3 themes regarding main experiences and triggers of MID. These sources of distress manifested both during and after the deployment (see Table 3 for additional quotations).

**Table 3.** Domains and Key Themes From Thematic Analysis of the Qualitative Data

Domain and theme	Description	Representative examples of respondents’ comments
Domain 1: distressing outcomes		
Horrifying injuries of war	Devastation of injuries	“I made 12 patients expectant. And I had them all driven to an area so they could die…And an hour later, all 12 of them died. So I was correct in all of those. But these are people who were able to look up to me and say, ‘Help me doctor.’ But I would look at him and say, ‘This is a war. Part of your skull is missing…Take this guy and put him in the expected pile.’” (participant 4)
		“There’d be an arm from one person, a leg from another person, part of their body, it’d be like seven people in a backpack and they’d say ‘Hey doc, you got to declare these people dead.’ Yeah. They’re dead. They’re definitely dead. It was just pieces of people.” (participant 2)
Patient population	Populations that were especially difficult for surgeons care for, including children, pregnant women, and US service members	“He died and that was pretty hard because everybody knew the soldier well and so there was a lot of distress and sadness amongst us. It was difficult because it’s a lot different from civilian trauma practice. Your patients are people you haven’t seen, haven’t met…but in this situation it was hard because everybody knew this man. I don’t know if I would say it was moral distress, but it was very difficult.” (participant 3)
		“It was very hard when I had a six-year-old that died on me, a six-year-old shouldn’t be shot so when they die, it’s just very difficult.” (participant 6)
Regret and second-guessing	Second-guessing clinical decisions or actions	“He was young…had isolated facial injury. There was nothing that would have killed him other than his airway. You think about things that you could have done differently…you think, we should have more securely tied down his tube or should have done an airway. Maybe then that wouldn’t have happened.” (participant 1)
Domain 2: medical rules of engagement		
Limited capabilities	Medical teams made smaller, more mobile, and closer to battlefront to decrease time to surgical care	“One of the things that happened is we kept getting divided into smaller and smaller teams and is because people think, well make the teams more mobile and be able to go further out, save more lives and I’ve been rebelling against this saying no, it doesn’t save more lives. All it does is put more people at risk.” (participant 16)
		“Now the push is for smaller and smaller teams to where on an Forward Surgical Team you would typically have two surgeons, two CRNAs. But now they’re splitting those to where it’s either two general surgeons or general surgeon, orthopedic surgeon, CRNA. So you really can only run at most two ORs at a time. A lot of times just one OR. And casualties rarely come in one at a time in these austere environments. If there’s a roadside bomb or an IED it’s multiple people at once so that’s always an issue. A lot of times you’ll have two patients come in who both need surgery so you gotta figure out how you temporize one while you operate on the other.” (participant 7)
Transfer to local facilities	Required to transfer local national casualties to local facilities that could not take care of acutely injured patients	“So you got a pretty sick patient that you can’t keep, you’ve done everything you can for them and you’re sending them essentially to certain death in some instances, if you transfer them out somewhere else. But that’s their whole local health care system. I’m not going to fix their whole health care system. So those are the challenges.” (participant 8)
		“The system prevented me from giving the care I wanted to give because obviously if you have a blown up five-year-old, you don’t necessarily want to put them in the back of a minivan and send them to the local hospital. You want to put them on an aircraft and get them to a well-resourced Western hospital.” (participant 1)
Issues with command	Command decisions made without taking into consideration resources	“This is probably most frustrating, is that they [command] would say, ‘You missed this injury.’ But we didn't see it until we saw it in the CT scan like you guys know we don’t have a CT scan, right? So there was a lot of Monday morning quarterbacking at the next station to where you know sometimes we would not always get the feedback that you did a good job or things people did well, you only hear really about the things that people criticize, even that sometimes was not warranted.” (participant 10)
		“It was a little bit crazy…I was really frustrated because they would claim they had two bed capability when they really only had two surgeons and one scrub tech and so that was a time where I really was frustrated with the top leadership and let them all know how ridiculous it was that they were claiming they had two surgical bed capability when they had like barely one.” (participant 17)
Domain 3: delayed personal impacts (effects of deployment after returning to US)		
Sleep	Sleep disturbances and nightmares	“Sleep deprivation is probably one of the bigger ones. Your schedule’s irregular, your clock is off. At one point, I remember they did a survey and it was like 60 to 70 percent of post-deployed service members were taking Ambien or something else for sleep pretty routinely. I’ve had team members who didn’t have access to it in the deployed environment but when they got home, they had problems with alcohol. Several of them had to seek treatment for that. Behavioral issues, coping issues, real deal PTSD.” (participant 8)
Interpersonal relationships	Feelings of disjointedness and difficulty with things that reminded them of deployment experiences, such as crowds, loud noises, and pressures	“You think I’m gonna get home and I’m just gonna be jumping for joy and be happy…. But the common experience I had and I found from others is you get home and for the first month or two, you’re just kind of numb. You’re not excited about much. You’re not sad about much. You’re just kind of numb and on cruise control and you don’t want to talk about much that happened.” (participant 5)
		“I had trouble coming back. Specifically, with crowds and that continues to this day…. That wasn’t an issue before deployed and I was never in crowds when I was deployed, but that bothers me. And then when I first got back, loud sounds specifically planes, helicopters, things like that because when the jets would fly over, that was always pretty overwhelming…. So loud sounds bothered me for a while when I got back. That doesn't really catch my attention anymore.” (participant 12)
Medical practice	Reintegration into surgical practice back in the US	“Actually when I got back in 2016 the very first case I did, I had probably the worst complication of my career. It was because I was rusty. Every time I deployed, I would not be on my A game when I came back and I probably shouldn’t have been doing this by myself as soon as I got back, even though I was only gone three months and I was clinically busy on my deployment. I wasn’t busy doing minimally invasive surgery on infants, so yeah, that was, that was that was hard.” (participant 18)

Abbreviations: CRNA, Certified Registered Nurse Anesthetist; CT, computed tomography; IED, improvised explosive device; OR, operating room; PTSD, posttraumatic stress disorder.

### Domain: Distressing Outcomes

#### Theme 1: Horrifying Injuries of War

All surgeons interviewed expressed substantial distress from witnessing horrific injuries, largely due to military weaponry and severe burn and blast injuries. One surgeon (participant 9) said, “The devastation of the injury cannot be duplicated by civilian trauma. Your trauma training and residency can’t match it no matter how bad it is. It’s just devastating. That shock takes a little bit to get over.”

Surgeons who were members of small surgical teams described feeling overwhelmed with the volume of casualties and perceived that they were frequently declaring patients as expectant or dead. The injuries they described as most horrifying and memorable were the blast injuries (“they just don’t make it and you just feel that sense of powerlessness” [participant 8]), burns (“worst complete 100% burns I’d ever seen on a human being” [participant 14]), and amputations (“devastating high amputations that happen in very, very young kids and to send them out to a devastated country with very little options for rehab and medical capabilities is tough” [participant 1]).

#### Theme 2: Patient Populations

Participants spoke about different patient populations in a war setting, and they often had the hardest time dealing with casualties involving children, pregnant women, and US soldiers. Just over half (11 of 20 surgeons) felt that treating US soldiers was particularly distressing because they felt connected to these patients in a deeper way. Some personally knew not only the soldiers themselves, but also their families. The families wanted surgeons “to assure them that we did everything we could and that their child didn’t die in agony, or despair and died around men and women who served with him and cared about him” (participant 8).

A majority of surgeons (16 of 20 surgeons) mentioned some memory or experience of treating children, and each of these experiences seemed to have made a substantial impact in their deployment experience overall. The experience was distressing not only because of the wartime injuries that these children sustained, but also because surgeons were not formally trained in pediatric surgery.

#### Theme 3: Second-guessing and Regret

A common (14 of 20 surgeons) feeling that many surgeons experienced during deployment was one of second-guessing and regretting some of the decisions that they were forced to make. One of the main reasons the surgeons gave for experiencing this distress was the fact that “a lot of these casualties in the United States, we would have treated and cared and could have recovered and they were basically just made expectant and given a morphine drip which is tough to get used to” (participant 10). Furthermore, surgeons said that they were not trained to do some operations that were required of them, which led to second-guessing of decisions made before and during surgical procedures. The surgeons also said that the limited resources, personnel, and poor outcomes experienced during the deployment also made them question the decisions they had to make and their own surgical abilities in very stressful situations.

### Domain: MROE

MROE are battlefield-specific rules governing the delivery of medical care to military personnel and local national civilians. Within the domain of MROE, the main sources of MID stemmed from transfer requirements of civilian patients to local facilities, limited capabilities, and disagreement with commanders.

#### Theme 1: Transfer Civilian Patients to Local Facilities

The most common source of distress was the MROE policy that required transfer of civilian casualties to local facilities that often could not provide the level of care needed (20 of 20 surgeons). Surgeons reported that they would assume or even know with certainty that their patients died after transfer. Some surgeons presumed their patients would die in these facilities whether by “being dumped in the river” (participant 1) or having the “trach tube pulled out” (participant 15) so that the facility could reuse it for a less sick patient their hospital. They reported that it was especially distressing watching the work and resources committed to the patient at their facility be for nothing when the patients were transferred to a facility that was unable to maintain the same level of care.

#### Theme 2: Limited Capabilities

The distance between the role 2 facilities and the commander dictating MROE led to frustration among surgeons, because they felt that command would expect the teams to continue to deliver the same care while shrinking their size so that they could fit into top-down strategy and “look good on paper” (participant 20). Surgeons described feeling upset that these small teams kept shrinking, to the point where they could no longer provide sufficient care because of limited supplies (mainly blood) that they could carry. One-quarter of the surgeons (5 of 20 surgeons) suggested that even consolidating 2 teams together could make for a much better facility that could accept more patients easily, but the military pushed for smaller teams to support all contingency forces. These austere conditions caused surgeons to have to conserve their blood supply or even stop midsurgery to save resources for more incoming patients.

Some of the surgeons (8 of 20 surgeons) expressed concerns about having to witness patients suffer because of strict protocols that did not apply to emergent medical situations presenting to treatment facilities: “They had so much security there…they had no idea the urgency of my business. When the patient would come, they wouldn’t let you get near the patient...Every Iraqi person was treated as hostile…The speed we were allowed to treat those people were as if…walking slowly over to a dying person. It’s absolutely maddening…you can’t go and touch the patients without some Marine screaming at you. That was a very bad experience. I was so glad to get out of there” (participant 4).

Moreover, most surgeons (14 of 20 surgeons) felt that they were unprepared for some of the specialized surgical procedures they would have to perform, mainly thoracic and pediatric procedures. Several surgeons mentioned that they did not have the necessary prior caseload experience to prepare them for deployment from their military hospital experience back home. They felt that the teams were not given enough time to prepare together before deployment and, often, they felt that the ancillary personnel were improperly or incompletely trained to support the operation, leading to substantial frustration and diminished patient outcomes.

#### Theme 3: Command Structure and Lack of Communication

The disagreement with and lack of communication from command teams located in higher level facilities made it so most surgeons (15 of 20 surgeons) could not ascertain the outcomes of US casualties who were transported to higher level facilities. Even if they were told eventually, it was distressing that the delay often made it too late to apply the lessons learned from those casualties into their practice.

Moreover, surgeons reported that the higher echelons of care would often criticize the clinical decisions made for US casualties treated by far-forward teams, resulting in a doubly distressing situation for surgeons in which they experienced MID not only from the loss of their patient or major complications, but from the criticism of command for things that they felt were outside of their control. In addition, “There were external forces telling us you need to give up [local civilian] care so you can treat the US people. At the same time, you need to treat the person in front of you, that’s your duty as a physician…. But if I’m going to treat the person in front of me, I need more resources so that I can treat the next person that comes through, because we’re only going out here with 20 units of blood” (participant 16). Surgeons acknowledged that this was often necessary in the setting of resource-limited facilities but expressed frustration in those choices when it they believed that commanders instituted these policies for the sole purpose of burnishing their outcomes for purposes of promotion.

### Individual-Level Effects of MID

MID in participants most commonly manifested in sleep disorders and problems with interpersonal relationships and medical practice. Some surgeons (7 of 20 surgeons) described bouts of insomnia during the deployment due to casualties coming in at night or owing to the stressful environment, resulting in extensive fatigue during deployment. Some (4 of 20 surgeons) discussed vivid nightmares after deployment: “I had recurring nightmares…The dream started where I was in the [operating room] and I’m sawing off the soldier’s arm on one side and the guys are sawing off the soldier’s arm on the other side... And when we take the arms off…I can just see a little tiny little hole and it didn’t need to be amputated. And then I would wake up. It was the same dream over and over again for months” (participant 10).

The most common manifestation of distress (14 of 20 surgeons) was emotional challenges that affected their ability to reintegrate. One participant (participant 17) stated, “One of the most stressful times is coming home. I was…surrounded by my friends. And you feel disjointed because they’re still talking about all the things that you used to talk about…and you just came from an environment where people were dying and there’s mortars going off…I don’t really fit in with this group anymore.”

They often did not want to speak about their deployments and saw questions as “ridiculous” or “inappropriate.” Talking to people who had similar deployment experiences was the most effective method of coping. Some participants (8 of 20 surgeons) described noticing everyday things affecting them, including big crowds, the national anthem, and loud noises eliciting anxiety or anger responses.

Six participants also described how the deployment affected their confidence in clinical practice once returning to the US. Many surgeons felt “rusty” and second-guessed themselves because they were not able to practice their specialized surgical procedures on deployment. They would even go as far as to ask colleagues to assist with cases that were routine for them before deployment: “We do feel rusty afterwards and you have to ask your partners to be around or have a look at something with you. And that’s real. I think that there have been cases where you wonder, is there something I could have or should have done differently? Was I rusty because I wasn’t doing this day in, day out like I would have otherwise been doing because I was deployed?” (participant 20).

## Discussion

To our knowledge, this pilot qualitative study is the first to characterize MID in combat surgeons during peak casualty periods. Military health care professionals face unique challenges resulting from their dual role as medical practitioners and military personnel. They must comply with all legal orders and do not have the option of leaving their duty station. The 2015 Defense Health Board Ethical Guidelines and Practices for US Military Medical Professionals recognizes the difficult balance of obligations to patients against obligations as military officers to help commanders maintain military readiness, as well as areas of ethical reservations or disagreement.^28^ Not surprisingly, a major source of MID found in this analysis centered around these issues.

The MMD-HP survey tool was developed for civilian health care, and survey statements were not designed to assess combat deployments in military health care environments and the unique stressors experienced in these settings. There are several other instruments used to assess MID in active-duty personnel or veterans (Moral Injury Events Scale, Moral Injury Questionnaire, and Moral Injury Symptom Scale–Military Version); however, these are centered around combat experience (eg, killing or violent acts) and, therefore, lack specific granularity to the military practice of medicine.^3,16,19^ The results of this study provide possible insight into similarities and differences between combat personnel and military surgeons. Both groups experienced MID due to war-associated violence with devastating injuries, second-guessing, and regret, coupled with similar individual impacts such as insomnia and difficulties reintegrating. Military surgeons had additional stressors, such as treatment of injuries, at times without specific training or experience, as well as transfer of treated civilian patients.

A military-specific MMD-HP survey did not exist at the time of this study. In 2019, the TriService Nursing Research Program received funding to adapt the MMD-HP to assess MID in TriService military critical care nurses. The adapted survey MMD-HP-M added 10 military environment–specific questions, such as “Being required to care for injured children despite inadequate preparation,” “Being unable to deliver quality care when my personal safety is at risk,” and “Experience compromised patient care owing to conflicting military regulations that limit health care delivery.”^29^ There has been a single validation study^29^ in active duty military nurses, where 22.4% of their survey population had not ever deployed, and even with this they demonstrated “moral distress associated with situations of moral responsibility in circumstances outside of what is expected” and found that MID was associated with a desire to leave military service. This survey once further refined and validated with a deployed military physician or surgeon cohort could represent an advance in MID characterization in this unique population.

### Limitations

There are several limitations to our study. First, the 20-surgeon sample size of this pilot study is small. We conducted interviews to thematic saturation to minimize this concern, but these results must be validated and extended into a larger population of deployed combat surgeons. In addition, there is a potential for recall bias based on the time interval from deployment and the interview. However, previous work^30^ shows that deployments are readily, vividly, and accurately recalled by service members regardless of time since deployment.

## Conclusions

Our study revealed specific contributors of MID in military surgeons not previously characterized. Thus, the MMD-HP, a tool designed for civilian health care, may not be applicable to the unique setting and practice of a combat surgeon. The most ubiquitous source of distress, the requirement to transfer civilian patients to local facilities, is specific to the military medical care as governed by MROE. The combination of stressors delineated by our study population, as well as serving in an active battle space, may result in MID specific to this group. This study offers insight for further innovations in efforts under way to adapt the MMD-HP tool for deployed military health care personnel and, in the future, could be used to develop improved tools for screening and intervention to improve the health and welfare of deployed combat surgeons.

## Notes

Supplement 1. **eTable 1.** US Military Casualties, Operation Iraqi Freedom (OIF) as of January 8, 2020 **eTable 2.** US Military Casualties, Operation Enduring Freedom (OEF) as of January 8, 2020

Supplement 2.
Data Sharing Statement

## References

[1] Litz BT, Stein N, Delaney E, . Moral injury and moral repair in war veterans: a preliminary model and intervention strategy. Clin Psychol Rev. 2009;29(8):695-706. doi:10.1016/j.cpr.2009.07.003 19683376

[2] Talbot SG, Dean W. Physicians aren’t “burning out.” They’re suffering from moral injury. STAT. July 26, 2018. Accessed December 17, 2019. https://www.statnews.com/2018/07/26/physicians-not-burning-out-they-are-suffering-moral-injury/

[3] Bryan CJ, Bryan AO, Anestis MD, . Measuring moral injury: psychometric properties of the moral injury events scale in two military samples. Assessment. 2016;23(5):557-570. doi:10.1177/1073191115590855 26092043

[4] Mason VM, Leslie G, Clark K, . Compassion fatigue, moral distress, and work engagement in surgical intensive care unit trauma nurses: a pilot study. Dimens Crit Care Nurs. 2014;33(4):215-225. doi:10.1097/DCC.0000000000000056 24895952

[5] Allen R, Judkins-Cohn T, deVelasco R, . Moral distress among healthcare professionals at a health system. JONAS Healthc Law Ethics Regul. 2013;15(3):111-118. doi:10.1097/NHL.0b013e3182a1bf33 23963112

[6] Barnes HA, Hurley RA, Taber KH. Moral injury and PTSD: often co-occurring yet mechanistically different. J Neuropsychiatry Clin Neurosci. 2019;31(2):A4-A103. doi:10.1176/appi.neuropsych.19020036 31012825

[7] Williamson V, Murphy D, Greenberg N. COVID-19 and experiences of moral injury in front-line key workers. Occup Med (Lond). 2020;70(5):317-319. doi:10.1093/occmed/kqaa052 32239155 PMC7184422

[8] Guttormson JL, Calkins K, McAndrew N, Fitzgerald J, Losurdo H, Loonsfoot D. Critical care nurse burnout, moral distress, and mental health during the COVID-19 pandemic: a United States survey. Heart Lung. 2022;55:127-133. doi:10.1016/j.hrtlng.2022.04.015 35561589 PMC9050623

[9] Epstein EG, Hamric AB. Moral distress, moral residue, and the crescendo effect. J Clin Ethics. 2009;20(4):330-342. doi:10.1086/JCE200920406 20120853

[10] Catlin A, Armigo C, Volat D, . Conscientious objection: a potential neonatal nursing response to care orders that cause suffering at the end of life? study of a concept. Neonatal Netw. 2008;27(2):101-108. doi:10.1891/0730-0832.27.2.101 18431964

[11] Wiegand DL, Funk M. Consequences of clinical situations that cause critical care nurses to experience moral distress. Nurs Ethics. 2012;19(4):479-487. doi:10.1177/0969733011429342 22619234

[12] Shepard A. Moral distress: a consequence of caring. Clin J Oncol Nurs. 2010;14(1):25-27. doi:10.1188/10.CJON.25-27 20118021

[13] Friedberg MW, Chen PG, Van Busum KR, . Factors affecting physician professional satisfaction and their implications for patient care, health systems, and health policy. Rand Health Q. 2014;3(4):1. 28083306 10.7249/rhq-03-04-01 PMC5051918

[14] Shale S. Moral injury and the COVID-19 pandemic: reframing what it is, who it affects and how care leaders can manage it. BMJ Leader. 2020;4(4):224-227. doi:10.1136/leader-2020-000295

[15] McKinney JM, Hirsch JK, Britton PC. PTSD symptoms and suicide risk in veterans: serial indirect effects via depression and anger. J Affect Disord. 2017;214:100-107. doi:10.1016/j.jad.2017.03.008 28288403

[16] Nash WP, Marino Carper TL, Mills MA, Au T, Goldsmith A, Litz BT. Psychometric evaluation of the Moral Injury Events Scale. Mil Med. 2013;178(6):646-652. doi:10.7205/MILMED-D-13-00017 23756071

[17] Steenkamp MM, Litz BT, Hoge CW, Marmar CR. Psychotherapy for military-related PTSD: a review of randomized clinical trials. JAMA. 2015;314(5):489-500. doi:10.1001/jama.2015.8370 26241600

[18] Steinert C, Hofmann M, Leichsenring F, Kruse J. The course of PTSD in naturalistic long-term studies: high variability of outcomes—a systematic review. Nord J Psychiatry. 2015;69(7):483-496. doi:10.3109/08039488.2015.1005023 25733025

[19] Koenig HG, Ames D, Youssef NA, . The Moral Injury Symptom Scale–Military Version. J Relig Health. 2018;57(1):249-265. doi:10.1007/s10943-017-0531-9 29196962

[20] Koenig HG, Boucher NA, Oliver RJP, . Rationale for spiritually oriented cognitive processing therapy for moral injury in active duty military and veterans with posttraumatic stress disorder. J Nerv Ment Dis. 2017;205(2):147-153. doi:10.1097/NMD.0000000000000554 28129259

[21] Östlund U, Kidd L, Wengström Y, Rowa-Dewar N. Combining qualitative and quantitative research within mixed method research designs: a methodological review. Int J Nurs Stud. 2011;48(3):369-383. doi:10.1016/j.ijnurstu.2010.10.005 21084086 PMC7094322

[22] Childers R, Parker P. The cost of deploying a role 2 medical asset to Afghanistan. Mil Med. 2015;180(11):1132-1134. doi:10.7205/MILMED-D-14-00640 26540703

[23] Defense Casualty Analysis System. DCAS reports: Operation Iraqi Freedom (OIF) casualty summary by month. January 8, 2020. Accessed January 9, 2020. https://dcas.dmdc.osd.mil/dcas/app/conflictCasualties/oif/byMonth

[24] Defense Casualty Analysis System. DCAS reports: Operation Enduring Freedom (OEF) casualty summary by month. January 8, 2020. Accessed January 9, 2020. https://dcas.dmdc.osd.mil/dcas/app/conflictCasualties/oef/byMonth

[25] Epstein EG, Whitehead PB, Prompahakul C, Thacker LR, Hamric AB. Enhancing understanding of moral distress: the measure of moral distress for health care professionals. AJOB Empir Bioeth. 2019;10(2):113-124. doi:10.1080/23294515.2019.1586008 31002584

[26] SocioCultural Research Consultants, LLC. Dedoose version 8.0.35. 2018. Accessed January 19, 2023. http://www.dedoose.com

[27] Mathison S. Encyclopedia of Evaluation. SAGE Publications; 2005. doi:10.4135/9781412950558

[28] US Defense Health Board. Ethical guidelines and practices for U.S. military medical professionals. March 3, 2015. Accessed January 19, 2023. https://www.health.mil/Reference-Center/Reports/2015/03/03/Ethical-Guidelines-and-Practices-for-US-Military-Medical-Professionals

[29] Wilson MA, Simmons A, Harris JI, . Adaptation and testing of a military version of the Measure of Moral Distress for Healthcare Professionals. Am J Crit Care. 2022;31(5):392-401. doi:10.4037/ajcc2022177 36045043

[30] Thome J, Terpou BA, McKinnon MC, Lanius RA. The neural correlates of trauma-related autobiographical memory in posttraumatic stress disorder: a meta-analysis. Depress Anxiety. 2020;37(4):321-345. doi:10.1002/da.22977 31815346

