# Supplementary material for: Characterizing Moral Injury and Distress in US Military Surgeons Deployed to Far-Forward Combat Environments in Afghanistan and Iraq
Source: JAMA Netw Open. 2023 Feb 23;6(2):e230484. doi: 10.1001/jamanetworkopen.2023.0484 (PMC9951040; doi:10.1001/jamanetworkopen.2023.0484)
Supplement: Supplement 1. — eTable 1. US Military Casualties, Operation Iraqi Freedom (OIF) as of January 8, 2020 eTable 2. US Military Casualties, Operation Enduring Freedom (OEF) as of January 8, 2020 [file jamanetwopen-e230484-s001.pdf]

Supplemental Online Content

Ryu MY, Martin MJ, Jin AH, Tabor HK, Wren SM. Characterizing moral injury and distress in US military surgeons deployed to far-forward combat environments in Afghanistan and Iraq. *JAMA Netw Open*. 2023;6(2):e230484. doi:10.1001/jamanetworkopen.2023.0484

eTable 1. US Military Casualties, Operation Iraqi Freedom (OIF) as of January 8, 2020

eTable 2. US Military Casualties, Operation Enduring Freedom (OEF) as of January 8, 2020

This supplemental material has been provided by the authors to give readers additional information about their work.

© 2023 Ryu MY et al. *JAMA Network Open.*

eTable 1. US Military Casualties, Operation Iraqi Freedom (OIF) as of January 8, 2020[23]

Year	TOTAL
2002	0
2003	2427
2004	8004
2005	5948
2006	6418
2007	6130
2008	2060
2009	688
2010	319
2011-2017	0
TOTALS	31994

* 2003 -2008 was used for this analysis

eTable 2. US Military Casualties, Operation Enduring Freedom (OEF) as of January 8, 2020[24]

Year	TOTAL
2001	33
2002	75
2003	100
2004	218
2005	271
2006	403
2007	752
2008	804
2009	2166
2010	5267
2011	5232
2012	2983
2013	1369
2014	457
2015-2020	18
TOTALS	20148

* 2009 -2012 was used for this analysis

© 2023 Ryu MY et al. *JAMA Network Open.*
