# Supplementary material for: Characterizing Moral Injury and Distress in US Military Surgeons Deployed to Far-Forward Combat Environments in Afghanistan and Iraq
Source: JAMA Netw Open. 2023 Feb 23;6(2):e230484. doi: 10.1001/jamanetworkopen.2023.0484 (PMC9951040; doi:10.1001/jamanetworkopen.2023.0484)
Supplement: Supplement 2. — Data Sharing Statement [file jamanetwopen-e230484-s002.pdf]

Data Sharing Statement

Ryu. Characterizing Moral Injury and Distress in US Military Surgeons Deployed to Far-Forward Combat Environments in Afghanistan and Iraq. *JAMA Netw Open*. Published February 23, 2023. doi:10.1001/jamanetworkopen.2023.0484

Data
Data available: No

Additional Information
Explanation for why data not available: Potential sharing agreements could be made by individual request.
